# Resource management with kernel-based approaches for grid-connected solar photovoltaic systems

**DOI:** 10.1016/j.heliyon.2021.e08609

**Published:** 2021-12-20

**Authors:** V.S. Bharath Kurukuru, Ahteshamul Haque, Mohammed Ali Khan, Frede Blaabjerg

**Affiliations:** a Advance Power Electronics Research Lab, Department of Electrical Engineering, Jamia Millia Islamia, New Delhi, India; b Department of Electrical Power Engineering, Faculty of Electrical Engineering and Communication, Brno University of Technology, Brno, Czech Republic; c Department of Energy, Aalborg University, Denmark

**Keywords:** Photovoltaic power, Smart inverters, Reactive power control, Kernels, Power loss, Voltage regulation

## Abstract

The increasing penetration of photovoltaic (PV) power generation into the distribution grids has resulted in frequent reverse active power flows, rapid fluctuations in voltage magnitudes, and power loss. To overcome these challenges, this paper identifies the resource management of grid-connected PV systems with active and reactive power injection capabilities using smart inverters. This approach is aimed to minimize the voltage deviations and power losses in the grid-connected systems to accommodate the high penetration of PV systems. A kernel-based approach is proposed to learn policies and evaluate the reactive power injections with smart inverters for improving grid profile, minimizing power losses, and maintaining safe operating voltage limits. The proposed approach performs inverter coordination through nonlinear control policies using anticipated scenarios for load and generation. To assess the performance of the proposed approach, numerical simulations are performed with a single-phase grid-connected PV system connected to an IEEE bus system. The results show the effectiveness of the proposed approach in minimizing power losses and achieving a good voltage regulation.

## 1 Introduction

Photovoltaics (PV) is considered as a logical solution to handle the drawbacks in conventional generation resources due to their local availability, falling prices, and sustainability. Nevertheless, the increasing share of renewable energy sources in the network is causing serious problems for the grid, such as reverse power flow, voltage fluctuation, etc. [1]. Moreover, these problems are caused due to the remote injection of renewable energy have strained the apparent power capabilities of substation transformers [2]. Besides, the fluctuations observed at the residential PV generations bring up the issue of uncertainty in generation depending mostly on climate and geographical location of the system. This has resulted in a highly dynamic and unpredictable real power generation. Thus, to avoid these fluctuations and have a stable grid operation, voltage regulation is required.

Traditionally, voltage regulation is carried out using different techniques like on-load tap changing (OLTC) in substation transformers, switching of capacitor banks, and step voltage regulators. In [3], [4], the issue of reactive power-sharing is solved via consensus-based distributed voltage control. Here, the developed voltage controller is combined with a conventional droop-based control technique for eliminating the line impedance mismatch. In [5], [6], a coordinated control strategy is proposed for reactive power injection with a grid integrated distributed generation (DG) system. This approach coordinates the DGs and controllable devices by constraining system variables under a prescribed operating condition. It is identified that these techniques critically challenge the reactive power control due to the increasing uncertainty in real power generation. Therefore, to alleviate these problems, the use of smart inverter technology with DG systems is widely adopted.

Traditionally, PV systems are interfaced with inverters primarily for MPPT and DC-AC conversion, and for achieving grid integration to form a DG system. In the present day scenario, these inverters are upgraded by interfacing them with advanced communication, metering, and control functionalities [7]. These inverters provide smart multi-unit control by regulating the real power limit, achieving ramp rate for real power limit, controlling reactive power output or power factor (PF), ride-through capability for specific grid disturbances, bi-directional power flow capability, and alternatives to conventional transfer trip schemes [7]. The use of smart inverters for reactive power control provides a fast responding solution for various grid objectives such as power loss minimization and voltage regulation [2]. In [8], a method to compensate voltage imbalance is performed by injecting active and reactive power control through the power conditioning system of inverters. Moreover, in [9], [10], [11], [12], [13], the coordinated control schemes are proposed for conserving voltage reduction in a smart inverter. The techniques coordinated the operations of automated volt-VAR controllers and aggregated the reactive power control. Further, with the increasing penetration level of PV power into the grid more sophisticated rules for interconnection are emerging too. Intelligent solutions to the problems present in the grid harnessing the inverter control capabilities will be the key for successful implementation of large-scale PV generation in the distribution grid [14]. In [15], a hierarchical coordinated volt-VAR optimization methodology is proposed. The issue related to multiple objectives has been addressed by implementing a fuzzy decision-making method and an *ε*-constraint method. These intelligent solutions incorporate a large range of control functions into newer PV inverter designs, which enhances the operation of the distribution grid [7].

Moreover, according to the amended IEEE 1547 standard [16], inverters are allowed to operate at non-unit PF, giving them the freedom to improve the grid voltage profile [17]. Besides, with the increasing number of inverters in the grid, it must be noted that the coordination of each inverter needs to be considered to achieve grid stability. Generally, the PV generation and instantaneous loads from any node in a typical distribution grid setup are communicated to a central utility controller [18]. This controller computes the reactive power injection set-points and communicates them to the inverters at different nodes to minimize the ohmic losses subject to voltage regulation constraints. At this instance, the utility controller has the task of identifying the optimal set-points for achieving reactive power injection for the inverters. This can be defined as an optimal power flow task, which is generally non-convex. In radial networks, this operation can be eased into a second-order cone program through polar coordinates [19], where the problem of power loss and voltage deviation minimization is solved. To alleviate the complexity of the involved optimization problems, approximate grid models have been employed in [20], [21], [22]. The reactive power control problem can be solved using centralized, decentralized, or local techniques [23], [24]. The centralized approaches need a good communication setup as global information is needed for control actions [25], [26], whereas, the decentralized methods require local or neighboring inputs for evaluating the control settings of single and unbalanced multiphase grids [27], [28]. The purely localized schemes provide reactive power support using only local measurements [29], [30]. Moreover, it is identified that the centralized schemes incur high computational complexity due to the communication of large datasets between the controller and each inverter [31]. Besides, the decentralized schemes exchange multiple communications among inverters [32], [33], and the local schemes have no guaranteed performance as the control setpoints depend only on the local inputs. This makes the system very unreliable for disturbances from other nodes. [34]. In [35], [36], the combination of central and local active/reactive power control was adapted for voltage regulation in DG systems. As seen from the prior works, most of the existing approaches either solve problems locally or centrally or through a combination of both while considering a linear decision rule on the input parameters. Besides, the control of smart inverters in the literature did not learn the input/output pairs of the inverter independently for achieving optimal power flow. Instead, they combined as a multi-function learning task by linearly relating to the optimal power flow problem. This formulation failed to yield a sparse control because of the voltage deviation at the inverter outputs. The significance of sparse control is to jointly learn the inverter rules by posing the optimal power flow problem as a multi-function learning task. This is considered as a resourceful representation of inverter control development as it saves the requirement for communication elements.

In light of these issues, this paper proposes a mapping of reactive power control approaches as linear or nonlinear policies concerning their input features. It is identified that the linear policies are restricted to capturing linear relations between the features and dependent variables, and very often can only capture second-order statistical relations. Such limitations call for extensions to nonlinear and higher-order algorithms. This is achieved by adapting kernel-based learning for modelling the reactive power control policies. The major contributions of this paper are:

• A decentralized approach is developed for evaluating the reactive power control policies for a voltage regulation constrained problem.
• The inverter coordination is performed through nonlinear control policies designed on a slower timescale using anticipated scenarios for load and generation.
• A kernel-based learning algorithm is utilized to evaluate the control policies on the basis of input scenario data.
• The kernel-based policies are modeled as a nonlinear function of input feature vector making it practically feasible for achieving the performance and complexity trade-off.

The remaining sections of the paper are organized as follows: Section 2 discusses the grid modeling to evaluate the reactive power dispatch in a radial network. Section 3 discusses various problems with the existing control models and identifies the shortcomings of different methods. The proposed kernel-based policies for reactive power control are discussed in section 4 and the numerical simulations are developed in section 5. The research is finally concluded in section 6.

## 2 Grid modelling

**Figure 1:**
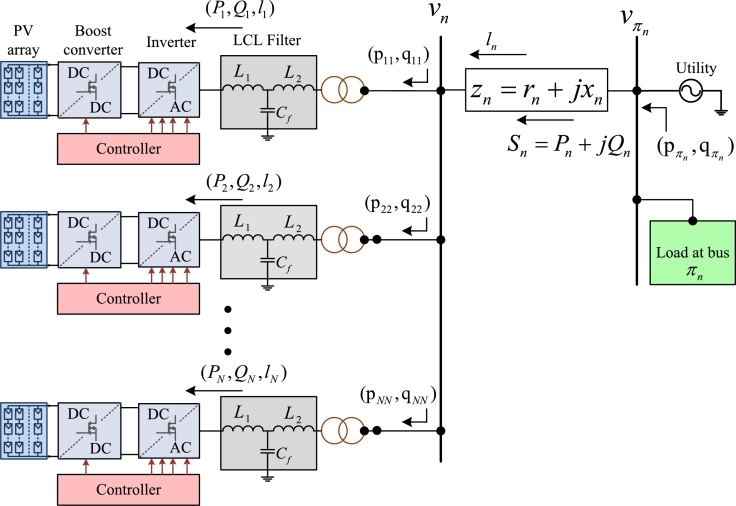
Distribution line *l*_*n*_ from parent bus *π*_*n*_ to bus *n*.

The grid-connected system is modeled by considering a radial single-phase grid with N+1 buses (indexed by n=1,…,N+1), and *M* branches. Generally, for a radial system with several branches M=N, every bus n=1,…,N is connected to a unique parent bus $\pi_{n}$ via distribution line shown in Fig. 1. Here, an approximated linearized distribution flow (LDF) model is used to evaluate the reactive power dispatch of the inverters. The grid is modeled by the branch flow equations given as [37]

(1) $$s_{n}=\sum_{k\in C_{n}}S_{k}-S_{n}+l_{n}\left(r_{n}+jx_{n}\right)$$

(2) vn=vπn−2Re[(rn−jxn)Sn]+ln(rn2+xn2)

(3) $$\left|S_{n}^{2}\right|=v_{\pi n}l_{n}$$

where for every line *n* the line impedance is $z_{n}=r_{n}+jx_{n}$, $l_{n}$ is the square of current magnitude in line *n*, $S_{n}=P_{n}+jQ_{n}$ is the complex power flow from the sending bus $\pi_{n}$ to bus *n*, $s_{n}=p_{n}+jq_{n}$ is the complex power injection at bus *n*, $v_{n}$ is the squared voltage magnitude at bus *n*, $C_{n}$ is the set of children buses for *n*, and the initial condition $s_{0}=\sum_{k\in C_{0}}S_{k}$. For all nodes n=1,…,N the real power injection is collected as a vector in $p:={\left[p_{1},\ldots ,p_{N}\right]}^{T}$, and the reactive power injection in $q:={\left[q_{1},\ldots ,q_{N}\right]}^{T}$, where these injections can be written as

(4) $$p=p^{g}-p^{c}$$

(5) $$q=q^{g}-q^{c}$$

where $p^{g}$ is the active power generation and the DG side, $p^{c}$ is the inelastic load power, and $q^{g}$ and $q^{c}$ are the reactive power injections at inverter, and load, respectively.

Moreover, the complex nodel injections are given by s=p+jq, and the squared voltage magnitudes are stacked as $v:={\left[v_{1},\ldots ,\;v_{N}\right]}^{T}$. Besides, all lines have resistance, reactance collected together as $r:={\left[r_{1},\ldots ,r_{N}\right]}^{T}$, and $x:={\left[x_{1},\ldots ,x_{N}\right]}^{T}$, respectively. The real and reactive line flows are defined as $P:={\left[P_{1},\ldots ,P_{N}\right]}^{T}$, and $Q:={\left[Q_{1},\ldots ,Q_{N}\right]}^{T}$, respectively, and the complex power flows are given as S=P+jQ. From (3) it is known that there exists a nonlinearity that complicates the power flow equations for the distribution line. Hence, to overcome this, the distribution grid is often remodeled as a linear model using the linear driving force (LDF) model [37]. As the line resistance, and reactance are small and their multiplication with squared current magnitude is less for evaluating the power flow equations at a flat voltage profile, the last summands in (1) and (2) can be dropped to formulate them as a linearized model.

The grid connectivity is captured in the branch-bus injection matrix $\overset{˜}{A}.\overset{˜}{A}\in {\left\{0,\;\pm 1\right\}}^{M\times (N+1)}$ and can be partitioned as $\overset{˜}{A}=\left[\begin{array}{cc} a_{0}\; & A \end{array}\right]$. This reduces the branch-bus injection matrix to A.A, which is an invertible square matrix $F:=A^{-1}$. Here, A follows

(6) $$a_{0}+A_{1}=0.$$

Using this connectivity matrix, the LDF can be rewritten as

(7) $$s=A^{T}S$$

(8) Av=2Re[dg(r−jX)S]−a0v0

where $v_{0}$ is the squared voltage magnitude at the substation.

Using (6) *S* can be eliminated from (7) and (8) giving the squared bus voltage magnitude for all buses n=1,…,N as [38]

(9) $$v≃2\mathrm{Rp}+2\mathrm{Xq}+v_{0}1_{N}$$

where

(10) $$R:=F^{T}\mathrm{dg}(r)F$$

(11) $$X:=F^{T}\mathrm{dg}(x)F$$

Since F≥0, the matrices R, X are also R≥0, X≥0. Moreover, by the properties of the matrices, it can be easily seen that R and X are symmetric positive definite with positive entries. Hence, bus voltages for all the buses in the grid are seen to increase if real or reactive power injections increase in the grid. Since losses have been ignored in (3), the squared voltage magnitudes are an overestimate concerning its original squared voltage magnitudes (9) with the bias depending on $l_{n}^{\prime }s$. But still, according to the numerical tests, the approximation errors in voltage magnitudes are seen to be less than 0.001p.u.

## 3 Problem formulation

Generally, the active and reactive power injections p,q can be decomposed into generation and inelastic load components as shown in (4) and (5). For known PV generation $p_{n}^{g}$ and to comply with its apparent power limit ${\bar{s}}_{n}^{g}$, the reactive power injected by inverter *n* is constrained through linear inequalities as

(12) $$\left|q_{n}^{g}\right|\leq {\bar{q}}_{n}^{g}:=\sqrt{{\left({\bar{s}}_{n}^{g}\right)}^{2}-{\left(p_{n}^{g}\right)}^{2}}.$$

Moreover, to cater to the voltage regulation in IEEE 1547 [16], a linear set of inequalities can be added.

(13) $$\underline{v}\leq v\leq \bar{v}$$

where $\underline{v}$, $\bar{v}$ are set according to the regulation guidelines and are usually taken as ±(3%−5%) about the nominal value [16]. To evaluate voltage deviations at each bus in the grid, let the sum of squared voltage magnitude deviations $\sum_{n=1}^{N}{\left(v_{n}-v_{0}\right)}^{2}$. Using the approximation in (9), and by ignoring the inconsequential scaling factor, the squared voltage deviations are

(14) $$\Delta_{s}\left(q^{g}\right):={\left\|R_{p}+X_{q}\right\|}_{2}^{2}.$$

Besides voltage deviation, the ohmic power losses are another critical quantity in the distribution grid operation. The active power losses can be expressed as $L=\sum_{n=1}^{N}r_{n}l_{n}$ or $\sum_{n=1}^{N}r_{n}\frac{P_{n}^{2}+Q_{n}^{2}}{v_{\pi n}}$. For small voltage deviations, as advocated in [2], the power losses (*L*) can be approximated as

(15) $$L=v_{0}^{-1}[P^{T}\mathrm{dg}\left(r\right)P+Q^{T}\mathrm{dg}(r)Q]$$

Using (10) and ignoring the inconsequential scaling by $v_{0}^{-1}≃1$, the power losses can be expressed as

(16) $$L=p^{T}\mathrm{Rp}+q^{T}\mathrm{Rq}$$

Since $p^{T}\mathrm{Rp}$ is a constant for a given set of data, the control variable q in the second summand in (16) is the function of interest. The power loss function can be expressed as

(17) $$L\left(q^{g}\right):=q^{T}\mathrm{Rq}.$$

For a positive definite matrix R, the positive convex quadratic function of $L(q^{g})$ is guaranteed. The objectives of voltage deviations $\Delta_{s}(q^{g})$ and power loss $L(q^{g})$ are contradicting in general. Thus, a multi-objective problem can be solved to cater to these contradictions. A convex combination of the objectives can be posed to formulate the reactive control optimization problem given as

(18) $$\min_{q^{g}}\lambda \Delta_{s}\left(q^{g}\right)+(1-\lambda )L(q^{g})\;s.t.\;q\in Q$$

where the set $Q\subseteq R^{N}$ captures the linear constraints in (12) for all n∈N. The voltage deviations and the ohmic losses are minimized by this formulation for different values of the parameter λ∈[0,1], related to the apparent power limit. It should be noted that in the above expression no voltage regulation limits apply, but the voltage deviation is minimized as a function of cost. To apply the voltage regulation limit, the problem can also be formulated as

(19) $$\min_{q^{g}}L\left(q^{g}\right)\;s.\mathrm{to}\;q\in Q,v\in V$$

where the set $V\subseteq R^{N}$ captures the linear constraints in (13) for all n∈N. Therefore, in this formula, the ohmic losses are kept to a minimum with respect to the apparent power limit and the voltage regulation limit.

## 4 Methodology

### 4.1 Control policies

To minimize net power loss and voltage drop of the network, the model should estimate the reactive power injected into each node. This injection depends on the size, layout, network topology and configuration of the inverter. In this study, the injection of reactive power $q_{n}^{g}$ by inverter *n* can be modeled as,

(20) $$q_{n}^{g}\left({\mathrm{zi}}_{n}\right)=f_{n}\left(z_{n}\right)+b_{n}$$

where the inputs ${\mathrm{zi}}_{n}$, $f_{n}$, and $b_{n}$ correspond to the controller input vector, controller function, and intercept, respectively.

***Controller inputs:*** Vector ${\mathrm{zi}}_{n}\in ZI_{n}\subseteq R^{M_{n}}$ is given as an input to the inverter to evaluate the reactive power injection at node *n*. This purely depends on its local values (${\mathrm{zi}}_{n}:={\left[\begin{array}{ccc} p_{n}^{g}-p_{n}^{c}\; & {\bar{q}}_{n}^{g}\; & q_{n}^{c} \end{array}\right]}^{T}$ where ${\bar{q}}_{n}^{g}:=\sqrt{{\left({\bar{s}}_{n}^{g}\right)}^{2}-{\left(p_{n}^{g}\right)}^{2}}$) or have few nonlocal or neighboring inputs like real power flow, squared magnitude. The entry of the local input ${\mathrm{zi}}_{n}$ is arranged in the following order: the active power input at node *n*, the maximum possible input of reactive power at node *n*, and the reactive load at this node. In addition, ${\mathrm{zi}}_{n}$ may be changed to comply with the management policy. This can be done by adding some important global inputs to the ${\mathrm{zi}}_{n}$ vector. It has been found in the literature that adding the square of the voltage value $v_{n}$ to ${\mathrm{zi}}_{n}$ will make it difficult to analyze the stability of the resulting closed-loop control, even if $f_{n}$ is linear [38], [39].

Since these inputs are available locally, there is a minimum burden on communication channels and the evaluation is quick. Ideally, if there are abundant communication resources, the uncertain quantities from all buses ${\left\{{\bar{q}}_{n}^{g},\;p_{n}^{c}-p_{n}^{g},q_{n}^{c}\right\}}_{n\in N}$ could be forwarded to all inverters. So, in this case the control input to ${\mathrm{zi}}_{n}$ is equal to and greater than all inverters in the grid. Also, the input variables are very flexible and can vary depending on the available network bandwidth. Non-local and common control inputs can be added to the input vector ${\mathrm{zi}}_{n}$ to study the results for the common local and global inputs of the inverter.

This gives ${\mathrm{zi}}_{n}:={\left[\begin{array}{cccccc} p_{n}^{g}-p_{n}^{c}\; & {\bar{q}}_{n}^{g}\; & q_{n}^{c}\; & P_{i}\; & P_{j}\; & P_{k} \end{array}\right]}^{T}$, where i,j, and *k* represent the line numbers, and $P_{i}$ is the real power flow on line *i*. These lines are selected on the bases of the topology of each network and these inputs will be identical to each inverter.

***Controller function:*** The next step includes the control function policy $f_{n}$. Control functions can be evaluated as linear or non-linear policies as defined below. Using kernel-based learning theory, the reactive power control of the inverter *n* is assumed to be in the Reproducing kernel Hilbert space (RKHS) [40].

(21) $$H\kappa_{n}:=\left\{f_{n}\left({\mathrm{zi}}_{n}\right)=\sum_{t=1}^{\infty }K_{n}\left({\mathrm{zi}}_{n},{\mathrm{zi}}_{n,t}\right)a_{n,t},a_{n,t}\in R\right\}$$

that is uniquely determined by the kernel function $K_{n}:ZI_{n}\times ZI_{n}\to R$. The linear policies can be implemented by evaluating a linear kernel $K_{n}\left({\mathrm{zi}}_{n,t},{\mathrm{zi}}_{n,t^{\prime }}\right)={\mathrm{zi}}_{n,t}^{T}{\mathrm{zi}}_{n,t^{\prime }}$ and the nonlinear policies can be designed by selecting a polynomial kernel $K_{n}\left({\mathrm{zi}}_{n,t},{\mathrm{zi}}_{n,t^{\prime }}\right)={\left({\mathrm{zi}}_{n,t}^{T}{\mathrm{zi}}_{n,t^{\prime }}+\gamma \right)}^{\beta }$, or a Gaussian kernel $K_{n}\left({\mathrm{zi}}_{n,t},{\mathrm{zi}}_{n,t^{\prime }}\right)=\exp\left(-{\left\|{\mathrm{zi}}_{n,t}-{\mathrm{zi}}_{n,t^{\prime }}\right\|}_{2}^{2}/\gamma \right)$ with design parameters *β* and γ>0, or by a linear combination of linear, polynomial, and gaussian kernels.

***Intercept:*** The control function also needs to evaluate an intercept value $b_{n}\in R$ in (20). Although it could be incorporated into $f_{n}$ by augmenting ${\mathrm{zi}}_{n}$ with a constant entry of 1, it is usually kept separate to avoid its penalization through $\left\|f\right\|\kappa_{n}$.

### 4.2 Learning policies from scenarios

The proposed approach deals with multiple generation units with multiple inverters that communicate with each other and communicate with the operator. In general, the process of data communication between different inverters establishes *N* inverter utility communication links and requires another *N* utility inverter communication links to communicate with the operator. This leads to traffic both between the operator and the inverter. To overcome this, operators can use a scenario sample approach. Instead of assessing the problem over a long period of time, the operator can decide to output the settings less frequently, for example every 10 minutes. After the control function and input vector are completed, the reactive power control policy (20) should be evaluated between the input data settings. Here, the $n^{th}$ entry of $q_{g}$ for any given scenario *t* can be replaced with the policy $q_{n}^{g}\left({\mathrm{zi}}_{n},t\right)=f_{n}\left({\mathrm{zi}}_{n},t\right)+b_{n}$ from (20). Thus, the algorithm evaluates the optimal function, and the intercept pair ${\left\{{\overset{ˆ}{f}}_{n},\;{\overset{ˆ}{b}}_{n}\right\}}_{n=1}^{N}$, which can be found via the functional minimization as

(22) $$\min\sum_{t=1}^{T}C\left(y_{t},{\left\{f_{n}\left({\mathrm{zi}}_{n,t}\right)\right\}}_{n},b\right)+\mu P\left(\left\{\left\|f_{n}\right\|\kappa_{n}\right\}\right)$$

(23) $$\mathrm{over}{\left\{f_{n}\in H\kappa_{n}\right\}}_{n=1}^{N},b$$

(24) $$\text{s.to }\left|f_{n}\left({\mathrm{zi}}_{n,t}\right)+b_{n}\right|\leq {\bar{q}}_{n,t}^{g},\forall n,t$$

(25) $${\underline{v}}_{n,t}\leq r_{n}\left(p_{n,t}^{g}-p_{n,t}^{c}\right)+x_{n}\left(f_{n}\left({\mathrm{zi}}_{n,t}\right)+b_{n}-q_{n,t}^{c}\right)\leq {\bar{v}}_{n,t},\forall n,t$$

where $b:={\left[b_{1},\ldots ,b_{N}\right]}^{T}$, constraint (24) represents the apparent power constraint, and (25) represents the voltage regulation constraint. The regularizer $P\left(\left\{\left\|f_{n}\right\|\kappa_{n}\right\}\right)$ has been added in (22) to avoid overfitting of control policies to scenario data.

The usual machine learning regression settings analyze the dependencies between feature data and target data and evaluate the closest fit. In this formulation, the network variables supplied to each controller serve as characteristic data and the reactive injection serves as the set value. Ideally, the designed function should work well with functional target pairs not found during the training or adaptation process. In a direct analogy, the control concept of an inverter is presented as a general task of adapting functions based on scenario data. Once you have designed features (policies), you can apply them to hidden feature data. The apparent power limit (24) applies to the training data, but the guidelines obtained by (22) (25) impose an apparent power limit of ${\mathrm{zi}}_{n,t^{\prime }}$'s with $t^{\prime }\notin \left\{1,\ldots ,\;T\right\}$ because the policy was trained only on the data up to scenario *T* where the limit is active. This limitation of kernel-based learning also occurs in scenario-based and random designs [41]. To overcome this limitation the reactive power evaluated at $t^{\prime }$ scenario for node *n* can be heuristically projected within $\left[-{\bar{q}}_{n,t^{\prime }}^{-g},\;+{\bar{q}}_{n,t^{\prime }}^{-g}\right]$ as

(26) $${\left[q_{n}^{g}\left({\mathrm{zi}}_{n,t^{\prime }}\right)\right]}_{{\bar{q}}_{n,t^{\prime }}^{g}}:=\max\left\{\min\left\{q_{n}^{g}\left({\mathrm{zi}}_{n,t^{\prime }}\right),{\bar{q}}_{n,t^{\prime }}^{g}\right\},-{\bar{q}}_{n,t^{\prime }}^{g}\right\}$$

Here, the optimal policies (functions) are evaluated individually for each inverter *n*. Therefore, the inverter policies are linked via the loss parameter *L*, since voltage deviation and power loss are affected by each feeder supplying reactive power. Similar multifunctional settings can be found in collaborative filtering or multitasking learning [42], [43]. In this study, a regularizer that can be divided into all inverters as shown in (27) [44] is adopted.

(27) $$P\left({\left\{\left\|f_{n}\right\|\kappa_{n}\right\}}_{n=1}^{N}\right)=\sum_{n=1}^{N}{\left\|f_{n}\right\|}^{2}\kappa_{n}$$

The well-known Representer Theorem [45] can be applied successively over *n* in (22)–(25). This ensures that

(28) $${\overset{ˆ}{f}}_{n}\left({\mathrm{zi}}_{n}\right)=\sum_{t=1}^{T}K_{n}\left({\mathrm{zi}}_{n},{\mathrm{zi}}_{n,t}\right){\overset{ˆ}{a}}_{n,t}$$

still holds for all *n*. Thus, after the optimal policies are evaluated, the coefficients ${\left\{{\overset{ˆ}{a}}_{n,t}\right\}}_{n,t}$, the control policies $\left\{{\overset{ˆ}{f}}_{n}\right\}$ can be evaluated at any other point. As seen earlier, the inverter policy ${\overset{ˆ}{f}}_{n}$ over the test data ${\left\{{\mathrm{zi}}_{n,t}\right\}}_{t=1}^{T}$ can be examined as

(29) $${\overset{ˆ}{f}}_{n}=K_{n}{\overset{ˆ}{a}}_{n},\;\forall n$$

where ${\left[K_{n}\right]}_{t,t^{\prime }}=K_{n}({\mathrm{zi}}_{n,t},{\mathrm{zi}}_{n,t^{\prime }})$ for $t,t^{\prime }\in \{1,\ldots ,T\}$, and ${\overset{ˆ}{a}}_{n}:={\left[{\overset{ˆ}{a}}_{n,1},\ldots ,{\overset{ˆ}{a}}_{n,T}\right]}^{T}$. Moreover, the regularizer term, the RKHS norms can be expressed as

(30) $${\left\|f_{n}\right\|}^{2}\kappa_{n}={\overset{ˆ}{a}}_{n}^{T}K_{n}{\overset{ˆ}{a}}_{n},\;\forall n$$

### 4.3 Optimal policy

*Voltage Drop and Power Loss Minimization:* After evaluating the control policy, it is necessary to evaluate the optimal ability to minimize voltage drop and power loss in the network. Thus, the problem of minimizing the ohmic loss in (27) and the regularizer (22) can be expressed as a linear bounded quadratic program.

Lemma 1 *If the data-fitting term in* (22) *is selected as*

(31) $$C\left(y_{t},{\left\{f_{n}\left({\mathrm{zi}}_{n,t}\right)\right\}}_{n},b\right)={\left\|Cq_{t}^{g}+y_{t}\right\|}_{2}^{2},\;t=1,\ldots ,T$$

*and the regularizing term as*

(32) $$P\left(\left\{\left\|f_{n}\right\|\kappa_{n}\right\}\right)=\sum_{n=1}^{N}{\left\|f_{n}\right\|}^{2}\kappa_{n}$$

*the functional optimization in* (22) *–* (25) *can be transformed into the vector minimization*

(33) $$\min\frac{1}{T}\left({\left\|\mathrm{CQ}+Y\right\|}_{F}^{2}+\mu \sum_{n=1}^{N}a_{n}^{T}K_{n}a_{n}\right)$$

(34) $$\mathrm{over}\;Q\in R^{N\times T},{\left\{a_{n}\in R^{T}\right\}}_{n=1}^{N},\;b\in R^{N}$$

(35) $$s.to\;Q^{T}=\left[K_{1}a_{1}+b_{1}1,\ldots ,K_{N}a_{N}+b_{N}1\right]$$

(36) $$-{\bar{q}}_{n}^{g}\leq K_{n}a_{n}+b_{n}1\leq {\bar{q}}_{n}^{g},\;\forall n$$

*where* $Y:=[y_{1},\ldots ,\;y_{T}]$ *and the entries of the vector* ${\bar{q}}_{n}^{g}:={\left[{\bar{q}}_{n,1}^{g},\ldots ,{\bar{q}}_{n,T}^{g}\right]}^{T}$ *have been defined in* (12) *.*

Proof of Lemma 1 Based on (20), and (29), the reactive power injection of inverter *n* for scenarios t=1,…,T can be expressed by the vector $K_{N}a_{N}+b_{N}1$. Then, the apparent power constraint for inverter *n* and across all scenarios can be expressed as given in (36). Considering the first summand in (33) and based on the equality in (35), the $t^{th}$ column of Q denoted by $q_{t}^{g}$ contains the reactive injections from all inverters for scenario *t*. The squared Frobenius norm of a matrix equals the sum of the squared $l_{2}$-norms of its columns, which follows $\sum_{t=1}^{T}{\left\|Cq_{t}^{g}+y_{t}\right\|}_{2}^{2}={\left\|\mathrm{CQ}+Y\right\|}_{F}^{2}$. Moreover, the second summand in (33) follows directly from (30). □

*Power Loss Minimization under Voltage Constraints:* The network implements a model to minimize power loss, taking into account the limitations of overall power and voltage regulation. This model focuses on keeping the voltage within specified limits. Therefore, after evaluating the control policy, the optimal function for minimizing the power loss associated with the constraint as described above is estimated. This problem can also be expressed in linear bounded quadratic programming.

Lemma 2 *If the data-fitting term in* (22) *is selected to minimize losses given as*

(37) $$C\left(y_{t},{\left\{f_{n}\left({\mathrm{zi}}_{n,t}\right)\right\}}_{n},b\right)={\left\|R^{\frac{1}{2}}\left(q_{t}^{g}-q_{t}^{c}\right)\right\|}_{2}^{2},\;t=1,\ldots ,T$$

*and the regularizing term as*

(38) $$P\left(\left\{\left\|f_{n}\right\|\kappa_{n}\right\}\right)=\sum_{n=1}^{N}{\left\|f_{n}\right\|}^{2}\kappa_{n}$$

*the functional optimization in* (22) *can be transformed into the vector minimization*

(39) $$\min\frac{1}{T}\left({\left\|R^{\frac{1}{2}}\left(Q-Q^{c}\right)\right\|}_{F}^{2}+\mu \sum_{n=1}^{N}a_{n}^{T}K_{n}a_{n}\right)$$

(40) $$\mathrm{over}\;Q\in R^{N\times T},{\left\{a_{n}\in R^{T}\right\}}_{n=1}^{N},\;b\in R^{N}$$

(41) $$s.to\;Q^{T}=\left[K_{1}a_{1}+b_{1}1,\ldots ,K_{N}a_{N}+b_{N}1\right]$$

(42) $$-{\bar{q}}_{n}^{g}\leq K_{n}a_{n}+b_{n}1\leq {\bar{q}}_{n}^{g},\;\forall n$$

(43) $$V=R\left(P^{g}-P^{c}\right)+X\left(Q-Q^{c}\right)+v_{0}1_{N\times T}$$

(44) $${\underline{v}}_{t}\leq v_{t}\leq {\bar{v}}_{t},\;\forall t$$

*where real and reactive power consumption vectors are stacked as columns of the* N×T *matrix* $P^{c}:=\left[p_{1}^{c},\ldots ,p_{T}^{c}\right]$ *, and* $Q^{c}:=\left[q_{1}^{c},\ldots ,q_{T}^{c}\right]$ *, respectively. The voltage at each bus are stacked as columns of the* N×T *matrix* $V:=\left[v_{1},\ldots ,v_{T}\right]$ *. Similarly, real power generation is* $P_{g}:=\left[p_{1}^{g},\ldots ,p_{T}^{g}\right]$ *and the entries of the vector* ${\underline{v}}_{t}:={\left[{\underline{v}}_{1},\ldots ,\;{\underline{v}}_{N}\right]}^{T}$ *,* ${\bar{v}}_{t}:={\left[{\bar{v}}_{1},\ldots ,{\bar{v}}_{N}\right]}^{T}$ *,* ${\bar{q}}_{n}^{g}:={\left[{\bar{q}}_{n,1}^{g},\ldots ,{\bar{q}}_{n,T}^{g}\right]}^{T}$ *where the limits for voltage regulation are defined in* (13) *, the reactive power has been defined in* (12) *.*

Proof of Lemma 2 Based on (20) and (29), the reactive power injection of inverter *n* for scenarios t=1,…,T can be expressed by the vector $K_{n}a_{n}+b_{n}1$. Then the limit of the apparent power for inverter *n* and all scenarios can be expressed as (42). In the LDF equation of (9), the voltage limit is expressed as (43). A linear limit is added for all *N* buses in all scenarios given in (44) to keep the voltage within certain limits. Considering the first term in (39) and based on the equation in (41), the $t^{th}$ column of Q as $q_{t}^{g}$ contains the reactive injection from all inverters for scenario *t*. The square of the Frobenius norm of a matrix is equal to the sum of the squares of the $l_{2}$-norm of the column which follows $\sum_{t}^{T}{\left\|R^{\frac{1}{2}}\left(q_{t}^{g}-q_{t}^{c}\right)\right\|}_{2}^{2}={\left\|R^{\frac{1}{2}}\left(Q-Q^{c}\right)\right\|}_{F}^{2}$. Moreover, the second summand in (39) follows directly from (30). The total cost is normalized by T. □

### 4.4 Implementing reactive control policies

After setting up the type of policy and regularizer for the problem, the reactive power control policies follow four steps. In the first step, the scenario data ${\left\{x_{t}\right\}}_{t=1}^{T}$ is created by the operator for all the scenarios from t=1,…,T. In the second step, the operator solves (33). In the third step, each inverter *n* receives the optimal policy coefficients $\left({\overset{ˆ}{a}}_{n},\;{\overset{ˆ}{b}}_{n}\right)$ and training data ${\left\{{\mathrm{zi}}_{n,t}\right\}}_{t=1}^{T}$ from the operator. For the final step, each inverter *n* collects the new $z_{n,t^{\prime }}$ and applies its projected control policy over the next *τ* mins.

(45) $${\left[q_{n}^{g}\left({\mathrm{zi}}_{n,t^{\prime }}\right)\right]}_{{\bar{q}}_{n,t^{\prime }}^{g}}={\left[\sum_{t=1}^{T}K_{n}\left({\mathrm{zi}}_{n,t^{\prime }},{\mathrm{zi}}_{n,t}\right){\overset{ˆ}{a}}_{n,t}+{\overset{ˆ}{b}}_{n}\right]}_{{\bar{q}}_{n,t^{\prime }}^{g}}$$

**Figure 2:**
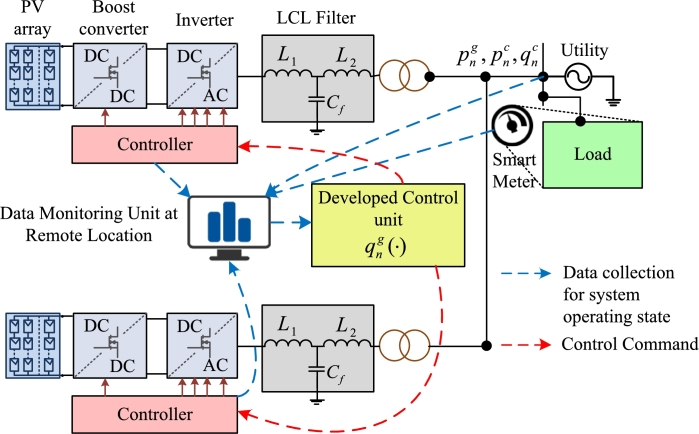
The proposed reactive power control methodology of a grid system with PVs.

Fig. 2 shows the proposed methodology divided into data collection, management rule development, and work steps. While developing control rules, operators collect input data from forecasts, feeder distortions and historical data. This data is collected and sorted as ${\left\{x_{t}\right\}}_{t=1}^{T}$. According to (33), the operator solves the problem of minimizing a quadratic plan as a linear constraint for policy studies. In the next step, the learned parameters $\left({\overset{ˆ}{a}}_{n},\;{\overset{ˆ}{b}}_{n}\right)$ and its training data ${\left\{{\mathrm{zi}}_{n,t}\right\}}_{t=1}^{T}$ are sent to each inverter *n*. This step is repeated every *τ* minutes depending on the needs of the power system and the availability of communication bandwidth. It is worth mentioning that if ${\mathrm{zi}}_{n,t}\in R^{M_{n}}$, the operator needs to send $\left(M_{n}+1\right)T+1$ data to inverter *n*. Besides, the number of scenarios *T* affects the amount of data being communicated to each inverter. As *T* increases, the bandwidth must be large to send data quickly. To do this, the inverter applies the acquired control policy to the parameters of the second stage. This recalls that all the learned parameters $\left({\overset{ˆ}{a}}_{n},\;{\overset{ˆ}{b}}_{n}\right)$ and ${\left\{{\mathrm{zi}}_{n,t}\right\}}_{t=1}^{T}$ are already readily available to each inverter *n*, and the fourth step can be iterated as compared to *τ* minutes of collecting data.

Also, for pure local control input ${\mathrm{zi}}_{n}$, control policy can be applied without additional information. In contrast, if the control inputs are not common or local, then a real-time record of inputs ${\mathrm{zi}}_{n,t}$ must be sent by the operator or origin before each inverter n. Also, broadcast protocols with broadband data can reduce communication overhead when input data is shared by inverters.

## 5 Numerical simulation

### 5.1 Simulation development

**Figure 3:**
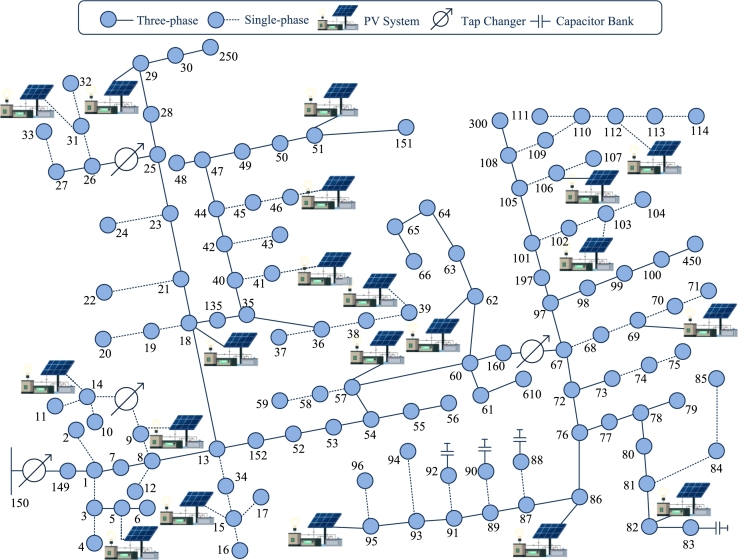
IEEE 123- bus benchmark feeder [47].

The rules developed for the reactive control of inverters were tested using the recommended IEEE 123 bus test feeders [46], [47] shown in Fig. 3, converted to a single-phase network using the procedure described in [48]. The node test feeder repository can be obtained from [49]. A 12.35kV base and a 100kVA power base was used. Residential load (actual power consumption) and PV module production data were generated based on a Gaussian mixture model for given mean and variance. The average values of actual power generation and power consumption were averaged $p_{g}^{mean}=2.5\;\text{kW}$, and $p_{c}^{mean}=10.25\;\text{kW}$, respectively. The variance *σ* was varied from (0−20)% in steps of 10%. The reactive load (the load with reactive power) was taken at a constant lagging power factor of 0.97. Analyzes were performed for 20%, 50%, and 100% PV penetration rates. Penetration rate is the ratio of solar powered buses to the total number of buses consuming energy. To compensate for reactive power, even in the presence of peak insolation, it was assumed that the inverter was made 10% larger to give a maximum power ${\bar{s}}_{n}^{g}=1.1{\bar{p}}_{n}^{g}$ for all *n*. The numerical analysis test involved five circuits. i) single power factor option where the inverter does not provide reactive power support; ii) the fixed Watt-VAR management rules detailed in [2]; iii) optimal reactive power setting [26], [50]; iv) the kernel-based approach of (33) and (39) to the linear kernel; and v) Gaussian kernel approaches to (33) and (39).

Kernel-based rules were learned using the load and generation data observed in the most recent T=10 scenario, and the parameters *μ* and *γ* were determined via 5-fold cross-validation [51]. Controller *n* was tested for $T^{\prime }=20$ different scenarios with only the local inputs (LI) ${\mathrm{zi}}_{n}={\left[{\bar{q}}_{n}^{g}p_{n}^{c}-p_{n}^{g}q_{n}^{c}\right]}^{T}$ for each *n*, with global inputs (GI) of power flows ${\mathrm{zi}}_{n}={\left[\begin{array}{cccc} {\bar{q}}_{n}^{g}p_{n}^{c}-p_{n}^{g}q_{n}^{c}\; & P_{15}\; & P_{16}\; & P_{17} \end{array}\right]}^{T}$, where lines 15, 16 and 17 were chosen as important lines dividing the grid into three separate branches at the first level. iii) A quadratic software operator partition solver was used to solve the v) method [52]. We initially estimated the cost $\lambda \Delta_{s}\left(q^{g}\right)+\left(1-\lambda \right)L(q^{g})$ for λ=1 to minimize the voltage deviation in the problem formula in (33). The results are then evaluated to minimize power loss and regulate the bus voltage. The cost $L(q^{g})$ is estimated according to the description of problem (39).

### 5.2 Tests for voltage drop minimization

To establish the problem, as shown in section 4.3, the voltage drop was minimized for the apparent power constraint as (33). Each T=10 test scenario was optimized by evaluating the optimal policy and tested against T=20 scenarios. Evaluated control policies were compared to 5 regimens. Circuit comparison and lack of response control are performed for Monte Carlo simulations. In this case, we extract the input vector ${\mathrm{zi}}_{n}$ from the previously described set of Gaussian distributions and then estimate the reactive power input for each circuit. At each start, the percentage improvement for the lack of reactive power support is estimated ($q^{g}=0$).

(46) $$\mathrm{improvement}\;\text{in}\;\mathrm{cost}\left(i\right)=\frac{1}{T}\sum_{t=1}^{T}\frac{C{|}_{q^{g}=0}-C{|}_{\mathrm{scheme}(i)}}{C{|}_{q^{g}=0}}\times 100\text{\%}$$

where $C{|}_{\mathrm{scheme}(i)}=\left({\left\|R_{p}+X_{q}\right\|}_{2}{|}_{\mathrm{scheme}(i)}\right)$ is the cost of the optimization, i∈{2,3,4,5} for each scheme as listed above. It should be noted that local control can perform much worse than a circuit without reactive support [2], which only evaluates reactive power control in terms of solar cell generation and local load. Therefore, to understand this condition, the reactive power control to minimize the voltage drop $\Delta_{s}\left(q^{g}\right)\lambda$ is set to 1, and the improvement rate for the insufficient reactive power support $q^{g}$ is regarded as 0. These results are listed in Table 1.

**Table 1:** Reactive power control for voltage drop minimization.

Network		Local (%)	Optimal (%)	Linear		Gaussian	
Penetration (%)	Variance (%)			Local Inputs (%)	Global Inputs (%)	Local Inputs (%)	Global Inputs (%)
20	10	4.47	99.51	95.93	98.8	95.99	99.29
20	20	74.25	99.98	96.7	99.31	97.77	99.42
50	20	88.46	99.99	97.74	99.66	97.95	99.62
50	20	90.92	99.99	92.12	99.5	93.1	99.56
100	20	69.65	99.99	94.67	99.16	94.86	99.25
100	20	96.35	99.99	96.12	99.1	97.04	99.21

Table 1 shows that the optimal control techniques outperform the other four methods. It evaluates the reactive power control to an optimal value with all inputs on all buses. This is true because optimal control works well as it solves the problem as a whole. The downside of this method is that the policy is slow and insecure with the communication overhead that requires high bandwidth to transmit data from every node to the substation at every moment. On the other hand, since local regulations estimate reactive power supply based on local input and take into account a constant x/r ratio for all lines, local regulations cannot control reactive control very accurately, resulting in very little improvement. Further, the proposed kernel policy provides a high percentage of improvement for both linear and non-linear policies. Also, the performance is very close to the optimal rule. As shown in Table 1, adding a global input (real power flow through lines 15, 16, and 17 in Fig. 3.) to the policy results in an estimated better reactive power delivery and a higher percentage improvement than using the local input alone.

**Figure 4:**
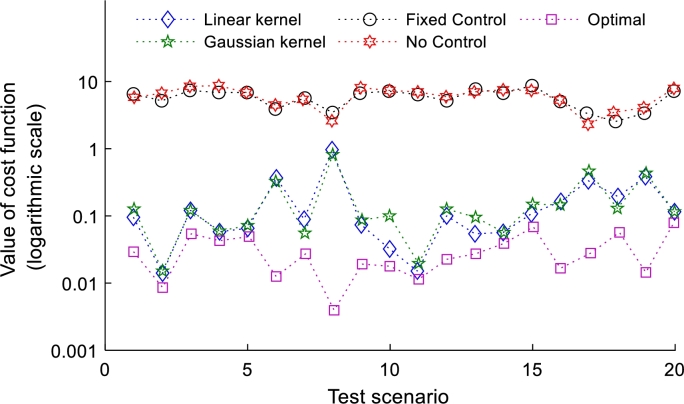
Reactive power control without global inputs for each scheme at 20% penetration, and 10% variance.

**Figure 5:**
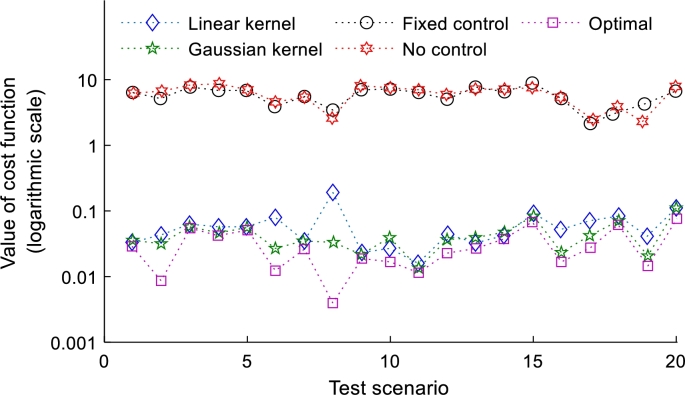
Reactive power control with global inputs for each scheme at 20% penetration, and 10% variance.

The voltage drop minimization test results are shown in Fig. 4, and 5. In the Fig. 4 shows the values of the log scale ${\left\|R_{p}+X_{q}\right\|}_{2}$ for each scenario. In this figure, it can be seen that local control behaves similar to or worse than the no reactive control scheme, while the linear and nonlinear policies proposed in this study behave very closely to the optimal control method. When comparing the results in Fig. 4, and Fig. 5 it is identified that with the addition of the global input data to the policy, the proposed method works much better and approaches optimal control. Adding these global inputs can increase data transfer and cyber overhead, but the increase will be small if the number of added inputs remains small. Only three features were added to this analysis, and a significant improvement in policy effectiveness was observed. Therefore, adding multiple global inputs can significantly improve the effectiveness of kernel policies. It is worth noting that the behavior of linear and nonlinear policies is very similar. These results encouraged to evaluate the power loss minimization using voltage regulation constraints, where networks are expected to behave differently for linear and non-linear policies.

### 5.3 Tests for power loss minimization under voltage constraints

The power loss minimization results with respect to voltage and apparent power constraints (39) are described below. Voltage violations between 3% of the base voltage value, i.e., $\underline{v}=0.97\;p.u$. and $\bar{v}=1.03\;p.u$. are allowed in this implementation. Each of the T=10 test scenario was optimized by evaluating the optimal policy and tested against T=20 scenarios. The evaluated control policies were compared against the five approaches previously described. For each scenario, the average voltage drop across the feeder was estimated as

(47) $$\Delta \bar{v}=\frac{{\left\|R_{p}+X_{q}\right\|}_{1}}{N}$$

**Figure 6:**
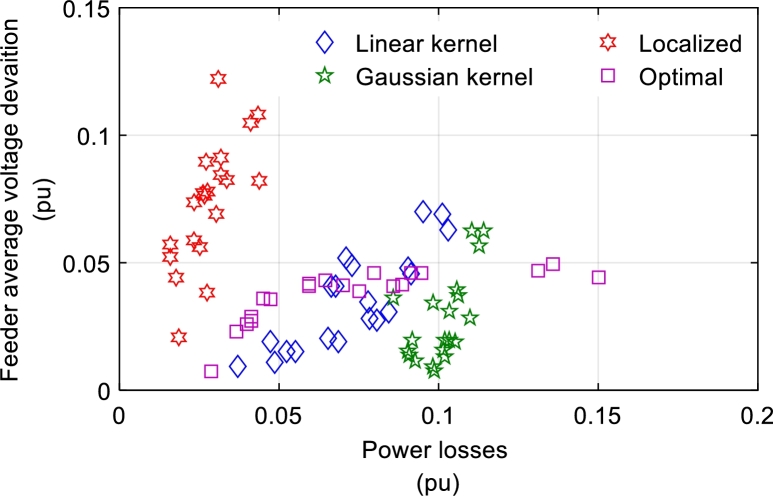
Average voltage-drop in a feeder for varying power loss under voltage constraints for different schemes at 100% penetration and 10% variance with local and global inputs.

The average voltage drop was compared to the reactive power loss. Fig. 6 shows the average voltage drop for each scheme with respect to 100% PV penetration and power loss, and 10% deviation in real power consumption. The local rule λ=0, minimizes the power loss of the voltage drop, regardless of the rule alone. However, it turns out that the voltage drop is very high, although the power loss is kept to a minimum. Therefore, local regulations cannot keep the voltage within the specified limits. Linear and nonlinear policies strictly follow the optimal control scheme. The model can violate the voltage constraints during testing because the voltage constraints are active only during the training step. As it is seen in the figure these violations are not very high on average.

**Figure 7:**
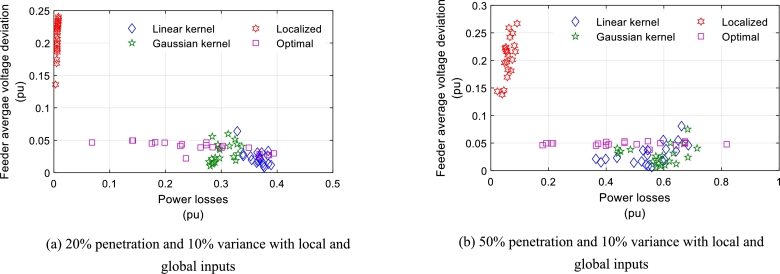
Average voltage-drop in a feeder for varying power loss under voltage constraints for different schemes.

From Fig. 6 it can be seen that for the global input, the average voltage-drop decreases and the model behaves closer to optimal. In this case, the non-linear policy provides better regulated voltage and lower power dissipation compared to the linear policy. From all the cases shown in Fig. 6, the non-linear policy has slightly more power dissipation, but the voltage regulation is better compared to the linear policy. A similar trend is shown in Fig. 7, which shows the average voltage drop of each circuit for power loss, solar device generation, and actual energy consumption deviation of 10% at 50% and 20% penetration of PV modules. The figure shows that the nonlinear policy works better than the linear one. Also, the average voltage of the bus has a lower voltage deviation from the rated voltage for the non-linear policy compared to the linear policy.

From the experiments and results, it is clear that local and optimal approaches solve problems locally or centrally, taking into account the linear rules of decision making on input parameters. It has also been found that using local inputs resulted in suboptimal results, whereas global inputs required complex computations. The core method developed from the distributed approach effectively evaluates reactive power control policies along with the problem of voltage regulation limiting. This makes the developed approach suitable for real-world implementation and allows you to find the right approach in terms of balance between performance and complexity. A comparative analysis of the existing flexible and developed approaches to the regulation of reactive power in distributed generation systems is presented in Table 2.

**Table 2:** Comparative analysis of flexible reactive power control approaches.

Method	Advantages	Limitations/Drawbacks	Remarks	Objective functions
Particle swarm optimization [14]	Less convergence time, Reduced problem solution space using depth first search, and prioritized loads during load shedding.	Reactive power control is not considered with the inverters in distribution generation system.	The optimization problem is formulated considering tree Knapsack problem. The load priority removes the least load in the 1st stage.	Least average principle to estimate the voltage deviation of selected nodes
Ant search algorithm [24]	Increased static stability in standalone mode operation.	Models reactive power control as a linear programming optimization problem, and works only with DC load Flow.	The algorithm convergence speed is increased using DC load flow approach.	Load shedding is minimized during standalone operation.
Adaptive optimization approach under frequency load shedding [18]	The load shedding problem is formulated as a mixed integer linear programming problem.	Complex power flow formulations that are not ideal for radial networks	An approximation of initial group AC operational limitation is considered with the optimization model during the islanding condition.	Based on location of load curtailments.
Kernel-based approach [Proposed]	Inverter coordination through nonlinear control policies using anticipated scenarios for load and generation. Minimizes power losses and achieves voltage regulation.	Voltage control inputs are not considered for varying controller inputs with the developed approach.	The proposed approach achieves desirable trade-off between reactive control performance and computational requirements	Linearly-constrained quadratic program

## 6 Conclusion

This paper developed a kernel-based reactive power control approach to achieve resource management and mitigating the impacts of varying loads and high PV penetrations in the distribution grid. In the developed approach, the policies have been designed to evaluate the control set-points for different scenarios and estimation has been done for the reactive power control in real-time using inputs and outputs individually. Besides, reactive power control policies are modeled by creatively cross-pollinating ideas from machine learning and using the powerful tool of kernel-based learning, which is practically feasible. Tests have been carried out for minimization of power losses and voltage regulation on an IEEE 123 bus system modeled as a single-phase grid. Compared to the techniques in the literature, the research results depicted are flexible and of adjustable nature. Further, this method can be extended for multistage formulations, varying controller inputs, and for evaluating the combination of kernels.

## Declarations

### Author contribution statement

V S Bharath Kurukuru: Conceived and designed the experiments; Performed the experiments; Wrote the paper. Ahteshamul Haque: Performed the experiments; Analyzed and interpreted the data; Wrote the paper. Mohammed Ali Khan & Frede Blaabjerg: Analyzed and interpreted the data; Contributed reagents, materials, analysis tools or data; Wrote the paper.

### Funding statement

This research did not receive any specific grant from funding agencies in the public, commercial, or not-for-profit sectors.

### Data availability statement

Data associated with this study has been deposited at the IEEE Power & Energy Society under the URL: http://sites.ieee.org/pes-testfeeders/files/2017/08/feeder123.zip.

### Declaration of interests statement

The authors declare no conflict of interest.

### Additional information

No additional information is available for this paper.
